# Development of an Ionic Liquid-Based Microwave-Assisted Method for the Extraction and Determination of Taxifolin in Different Parts of *Larix gmelinii*

**DOI:** 10.3390/molecules191219471

**Published:** 2014-11-25

**Authors:** Zaizhi Liu, Jia Jia, Fengli Chen, Fengjian Yang, Yuangang Zu, Lei Yang

**Affiliations:** 1 Key Laboratory of Forest Plant Ecology, Ministry of Education, Northeast Forestry University, Harbin 150040, China; E-Mails: zaizhiliu@hotmail.com (Z.L.); chenfengli@163.com (F.C.); zygorl@163.com (Y.Z.); 2 Pharmacy & Medical Laboratory Department, Daqing Medical College, Daqing 163312, China; E-Mail: jiajiaplay2006@163.com

**Keywords:** taxifolin, *Larix gmelinii*, ionic liquid, microwave-assisted extraction, distribution

## Abstract

An ionic liquid-based microwave-assisted extraction method (ILMAE) was successfully applied for the extraction of taxifolin from *Larix gmelinii*. Different kinds of 1-alkyl-3-methylimidazolium ionic liquids with different kinds of cations and anions were studied and 1-butyl-3-methylimidazolium bromide was chosen as the optimal solvent for taxifolin extraction. The optimal conditions of ILMAE were determined by single factor experiments and Box-Behnken design as follows: [C_4_mim]Br concentration of 1.00 M, soaking time of 2 h, liquid-solid ratio of 15:1 mL/g, microwave irradiation power of 406 W, microwave irradiation time of 14 min. No degradation of taxifolin had been observed under the optimum conditions as evidenced from the stability studies performed with standard taxifolin. Compared with traditional solvent and methods, ILMAE provided higher extraction yield, lower energy and time consumption. The distribution of taxifolin in different parts of larch and the influences of age, orientation, and season on the accumulation of taxifolin were analyzed for the sufficient utilization of *L. gmelinii*.

## 1. Introduction

*Larix gmelinii* (larch) is a medium-sized deciduous coniferous tree mainly found distributed in the Hinggan Mountains of China, North Sakhalin, and East Siberia [1]. At present, the amount of *L. gmelinii* is the largest among the other tree species of China. Due to its particular physical characteristics, such as rigidness, straight grain and corrosion resistance, larch has been widely applied to building and furniture manufacture and as a result large amounts of side products (logging slashes, bucking residues, and processing residues) are produced every year. Hence, much attention should be paid to the comprehensive utilization of larch resources. Recent studies have reported that two bioactive compounds—taxifolin and arabinogalactan—exist in *L. gmelinii* [2,3]. Compared with other plant sources of taxifolin such as *Rosa davurica* [4], *Engelhardtia roxburghiana* [5] and *Silybum marianum* [6], *L. gmelinii* accounts for a large proportion mainly because the timber yield and taxifolin content of *L. gmelinii* are much larger than that of other plants. While current studies are mainly focused on extracting taxifolin from larch wood, systematic researches of this compound in larch are nonexistent.

**Figure 1:**
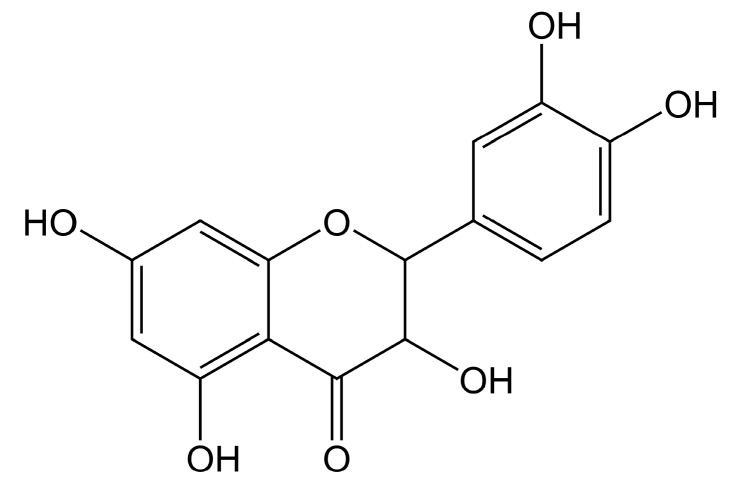
The molecular structure of taxifolin.

Taxifolin (3,3',4',5,7-pentahydroxiflavanone, Figure 1), also known as dihydroquercetin, is a bioactive component (flavanonol) [7,8]. As reported, taxifolin is widely used in the pharmaceutical industry as it can prevent diabetic cardiomyopathy [9], enhance mitotic arrest and apoptosis [10], suppress UV-induced skin carcinogenesis [11], act as type I inhibitor for VEGFR-2 kinase [12], and inhibit reductase [13]. It also has been used as a kind of natural antioxidant additive in the food industry [8].

Several extraction methods have been applied to extract taxifolin, which include heating, boiling or refluxing extraction with water and organic solvents [14,15,16]. However, there are many disadvantages in traditional extraction methods, such as being highly time-consuming and energy-consuming, low product recovery, tedious work-up procedures, and high consumption of organic solvents, which are often flammable and toxic, and responsible for the greenhouse effect. Thus, the development of a safe and environmentally benign extraction process is becoming increasingly necessary and important for the procedures of sample preparation and analytical determination. Compared with traditional extraction methods, the microwave-assisted extraction method has been widely used because it is more convenient, less time consuming and has higher efficiency for the extraction of bioactive compounds from plant materials [17,18,19,20,21].

Room temperature ionic liquids, also known as molten salts with a melting point fixed at ambient temperature or below 100 °C, are made up of organic cations and inorganic or organic anions [22]. Due to their particular characteristics, such as negligible vapor pressure, thermal and chemical stability, wide liquid range, no inflammability, and no ignition point [23,24,25], ionic liquids have been successfully used in the separation of bioactive substances, such as lignans [17,22], coumarins [23], glycosides [25], triterpenoids [24], flavonoids [18,24], procyanidins [26], alkaloids [27,28,29] and organic acids [30,31,32]. As ionic liquids can effectively absorb microwave energy, adding ionic liquids to an extraction system can improve the extraction efficiency [33]. In addition, the proposed ionic liquid-based microwave-assisted extraction (ILMAE) method could be regarded as a promising method for green extraction as reported in previous literature [34]. ILMAE is an innovative method, which uses an aqueous solution of ionic liquids as extraction solvent, reduces energy consumption and unit operations, and it is safer than the use of typical organic solvents and avoids denaturation of bioactive substances. Hence, it is very meaningful to discuss microwave-assisted extraction of taxifolin using ionic liquid aqueous solutions.

The aim of the present study is: (i) development a rapid and effective ionic liquid-based microwave-assisted extraction approach for the extraction of taxifolin from larch wood. Herein we describe our investigations of the performance of various ionic liquids with different cations and anions in the ILMAE method. Water stirring extraction (WSE), water reflux extraction (WRE), and maceration extraction (ME) were studied and compared with ILMAE. Meanwhile, the proposed method was validated with regard to stability, repeatability, and recovery experiments; (ii) the distribution of taxifolin in different parts of larch and the influences of age, orientation, and season on taxifolin accumulation were investigated to provide basic data for the further utilization of larch resources.

## 2. Results and Discussion

### 2.1. Screening of the Ionic Liquid-Based Extraction Solvents

The extraction yield of target compounds might be obviously affected by the physical and chemical properties of ionic liquids, while the two properties can be significantly influenced by their structure [29]. To find the optimal ionic liquid for taxifolin extraction and evaluate its influence on the microwave-assisted extraction process, 1-alkyl-3-methylimidazolium-type ionic liquids with different anions and cations were researched.

#### 2.1.1. Influence of the Anion

**Figure 2:**
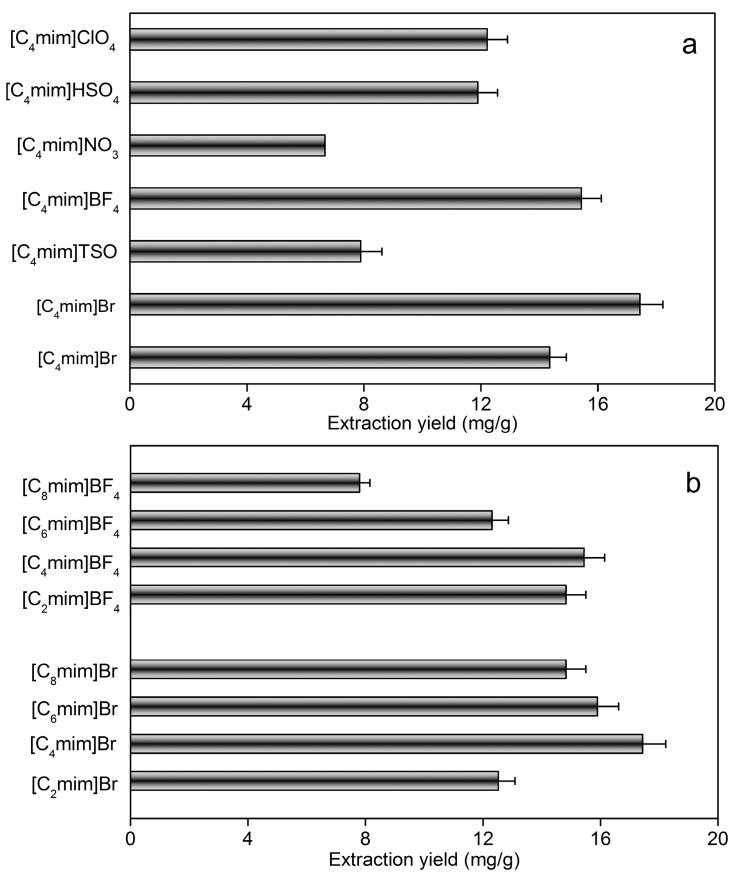
Influences of ionic liquid anion and cation on the extraction yield of taxifolin. (**a**) 0.5 g of dried sample was mixed with 10 mL 1.00 M ionic liquids of different anions and then soaked for 2.0 h, the suspension was extracted 10 min at 385 W with ILMAE; (**b**) 0.5 g of dried samplewas mixed with 10 mL 1.00 M ionic liquids of different cations and then soaked for 2.0 h, the suspension was extracted 10 min at 385 W with ILMAE.

The anion identity is considered an obvious factor which can influence the characteristic of ionic liquids, especially for water miscibility [35]. Therefore, N-methylimidazolium based ionic liquids with simple anions (Cl^−^, Br^−^) and complex anions (BF_4_^−^, NO_3_^−^, TSO^−^(TSO = tosylate), HSO_4_^−^ and CIO_4_^−^) were evaluated. The extraction yields of taxifolin were obviously different, as shown in Figure 2a. All of the selected ionic liquids were hydrophilic enough to dissolve in any proportion with water. The results indicated that ionic liquids with BF_4_^−^ and Br^−^ anions were more efficient for the extraction of taxifolin (Br^−^ being the most efficient). It also confirmed that the taxifolin extraction yield is anion-dependent.

#### 2.1.2. Influence of Cation

Eight ionic liquids with different cations (C_2_mim^+^, C_4_mim^+^, C_6_mim^+^, and C_8_mim^+^) combined with Br^−^ or BF_4_^−^ were also screened to obtain the optimal extraction yield of taxifolin. The results are shown in Figure 2b. They indicated that for ionic liquids linked with either Br^−^ or BF_4_^−^, the extraction yield of taxifolin first increased with the increasing alkyl chain length from ethyl to butyl, and then decreased with the alkyl chain length of the cation increasing from butyl to octyl. Consideration these effects on taxifolin extraction, [C_4_mim]Br was selected as the optimal extraction solvent for the subsequent extraction parameter optimization studies.

### 2.2. Optimization of Single Factor Extraction Conditions

#### 2.2.1. Influence of [C_4_mim]Br Concentration

**Figure 3:**
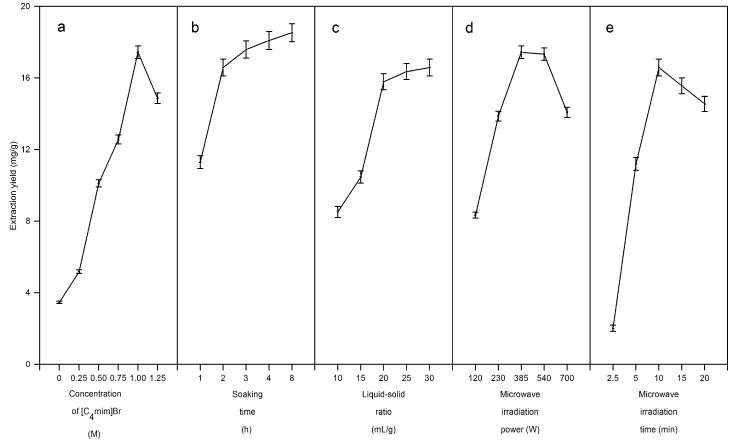
Single factor experiment. (**a**) Influences of [C_4_mim]Br concentration(0 M, 0.25 M, 0.50 M, 0.75 M, 1.00 M, and 1.25 M); (**b**)soaking time (1 h, 2 h, 3 h, 4 h, and 8 h); (**c**) liquid-solid ratio (10 mL/g, 15 mL/g, 20 mL/g, 25 mL/g, and 30 mL/g); (**d**) microwave irradiation power (120 W, 230 W, 385 W, 540 W, and 700 W); (**e**)microwave irradiation time(2.5 min, 5 min, 10 min, 15 min, and 20 min).

Experiments were carried out with different concentrations (from 0.25 to 1.25 M) to determine the optimum [C_4_mim]Br concentration for the microwave-assisted extraction of taxifolin. As shown in Figure 3a, the extraction yield of taxifolin increased with increasing [C_4_mim]Br concentration from 0.25 to 1.00 M. However, when the concentration reached 1.25 M, the taxifolin extraction yield decreased instead. This is because both the solubility of the target compound in the solvent and the microwave absorption capacity of the ionic liquids were improved with the increasing concentration, but a high concentration of ionic liquid resulted in high viscosity, which is not good for the penetration of the solvent into the plant tissue and also causes high ionic liquid consumption, so 1.00 M [C_4_mim]Br was selected as the optimal ionic liquid concentration.

#### 2.2.2. Influence of Soaking Time

To extract target compounds from the cellular structure, a solvent must have access to the cellular compartments of the target compounds. For dry larch wood powder, sufficient soaking time is indispensable to absorb sufficient microwave energy during the extraction process. Wood powder (0.5 g) was soaked in 1.00 M [C_4_mim]Br (10 mL) for 1, 2, 3, 4 or 8 h at room temperature before microwave irradiation. As shown in Figure 3b, the extraction yield of taxifolin apparently increased as the soaking time increased from 0 to 2 h, and then increased slowly with the longer soaking times (from 4 to 8 h). Therefore, 2 h was selected as the optimal soaking time.

#### 2.2.3. Influence of Liquid-Solid Ratio

Liquid-solid ratio, as an important parameter in the extraction process, was also evaluated for optimization. Large solvent volumes may lead to complicated extraction processes and unnecessary waste, while small volumes may make the extraction procedure incomplete. Thus, a series of experiments were carried out with different liquid-solid ratios (10:1, 15:1, 20:1, 25:1, and 30:1 mL/g) to evaluate the effect of liquid-solid ratio on the extraction yield. As shown in Figure 3c, the extraction yield apparently increased with increasing solvent volume up to 20:1 mL/g. With a higher liquid-solid ratio, greater contact between larch wood powder and [C_4_mim]Br aqueous solution occurred and a larger amount of taxifolin was finally extracted. When the liquid-solid ratio was changed from 20:1 to 30:1 mL/g, the higher solvent volume did not evidently improve the extraction yield. For commercial applications, a liquid-solid ratio between 15:1 and 25:1 mL/g should be optimized to avoid waste of solvent in the subsequent processes.

#### 2.2.4. Influence of Microwave Irradiation Power

Extractions were performed with 1.00 M [C_4_mim]Br at different microwave irradiation powers (120, 230, 385, 540 and 700 W). As shown in Figure 3d, the extraction yield of taxifolin obviously increased as the microwave irradiation power increased from 120 to 385 W and then decreased slightly as the microwave irradiation power changed from 385 to 540 W, while it decreased rapidly when the microwave irradiation power was higher than 540 W. This is because the ionic liquid has a strong ability to absorb microwave energy, which may result in scorching of the plant samples and thus the extraction yield of taxifolin decreased. However, low microwave irradiation power will take a long period of time to extract the target analytes. Therefore, 230–540 W was selected as the range of microwave power for subsequent experiments.

#### 2.2.5. Influence of Microwave Irradiation Time

Several experiments were carried out at 385 W with different microwave irradiation times (2.5, 5, 10, 15 and 20 min, respectively). Figure 3e indicates that the extraction yield of taxifolin increased rapidly as the microwave irradiation time increased from 2.5 to 10 min, while changing the microwave irradiation time from 10 to 20 min resulted in a decreased extraction yield. The low extraction yield of taxifolin obtained from the first 5 min demonstrated that microwaves need time to disrupt the cell walls of samples and to assist with the release of taxifolin into the solvent, but long microwave irradiation times (15 and 20 min) did not result in obvious improvements of the extraction yield. It also showed that taxifolin was mainly extracted from larch wood powder in the first 10 min during the whole extraction process. Based on these results, 5–15 min microwave irradiation time was selected for the following experiments.

### 2.3. Further Optimization by Response Surface Methodology (RSM)

RSM is an effective analysis technique which can be applied to optimize a process and thus obtain a satisfactory extraction yield. This method employs quantitative data from a series of designed experiments to analyze and simultaneously solve polynomial quadratic equations through regression analysis, which focuses on the significant factors and their relations, builds an empirical model, and thus optimizes the condition of factors for obtaining satisfactory response values. In order to find the optimum values for the different experimental variables and, also, to investigate the interactions between them, microwave irradiation time, liquid-solid ratio, and microwave irradiation power were optimized by RSM. From Table 1 and Table 2, the Model F-value of 29.21 implies the model is significant. *X_1_*, *X_3_*, *X_2_ X_3_*, *X_1_*^2^, *X_3_*^2^ are significant due to the “Prob > F” values of these model terms were less than 0.0500. The “Lack of fit F-value” of 0.93 implies the Lack of fit is not significant relative to the pure error. As shown in Table 3, the standard deviation of the model is 0.4. The “Pred *R^2^*” of 0.8025 is in reasonable agreement with the “Adj *R^2^*” of 0.9407. The ratio of 17.440 (higher than 4) indicates an adequate signal and thus this model can be used to navigate the design space. The extraction yield of taxifolin was given by the following equation:

*Y* = −39.09 + 1.12*X_1_* + 0.23 *X_3_* − 1.60 × 10^−3^*X_2_ X_3_* − 0.03 *X_1_^2^* − 2.42 × 10^−^^4^*X_3_^2^* (1)

**Table 1:** Experimental design matrix to screen for variables that determine the extraction yield of taxifolin from larch.

Run	Factor *X_1_*	Factor *X_2_*	Factor *X_3_*	Response	
	Microwave Irradiation Time (min)	Liquid-Solid Ratio (mL/g)	Microwave Irradiation Power (W)	Actual Extraction Yield (mg/g)	Predicted Extraction Yield(mg/g)
1	10 (0)	15 (−1)	540 (1)	16.69	16.68
2	10 (0)	15 (−1)	230 (−1)	14.47	14.16
3	10 (0)	25 (1)	230 (−1)	16.05	16.06
4	15 (1)	25 (1)	385 (0)	18.47	17.96
5	5 (−1)	25 (1)	385 (0)	16.89	16.83
6	15 (1)	20 (0)	230 (−1)	15.26	15.50
7	15 (1)	15 (−1)	385 (0)	18.37	18.44
8	15 (1)	20 (0)	540 (1)	15.95	15.90
9	5 (−1)	15 (−1)	385 (0)	15.80	16.06
10	10 (0)	20 (0)	385 (0)	18.47	18.21
11	5 (−1)	20 (0)	540 (1)	14.75	14.51
12	10 (0)	20 (0)	385 (0)	18.18	17.96
13	10 (0)	20 (0)	385 (0)	17.48	17.96
14	10 (0)	20 (0)	385 (0)	17.63	17.96
15	10 (0)	20 (0)	385 (0)	18.02	17.96
16	10 (0)	25 (1)	540 (1)	15.01	15.32
17	5 (−1)	20 (0)	230 (−1)	13.08	13.13

**Figure 4:**
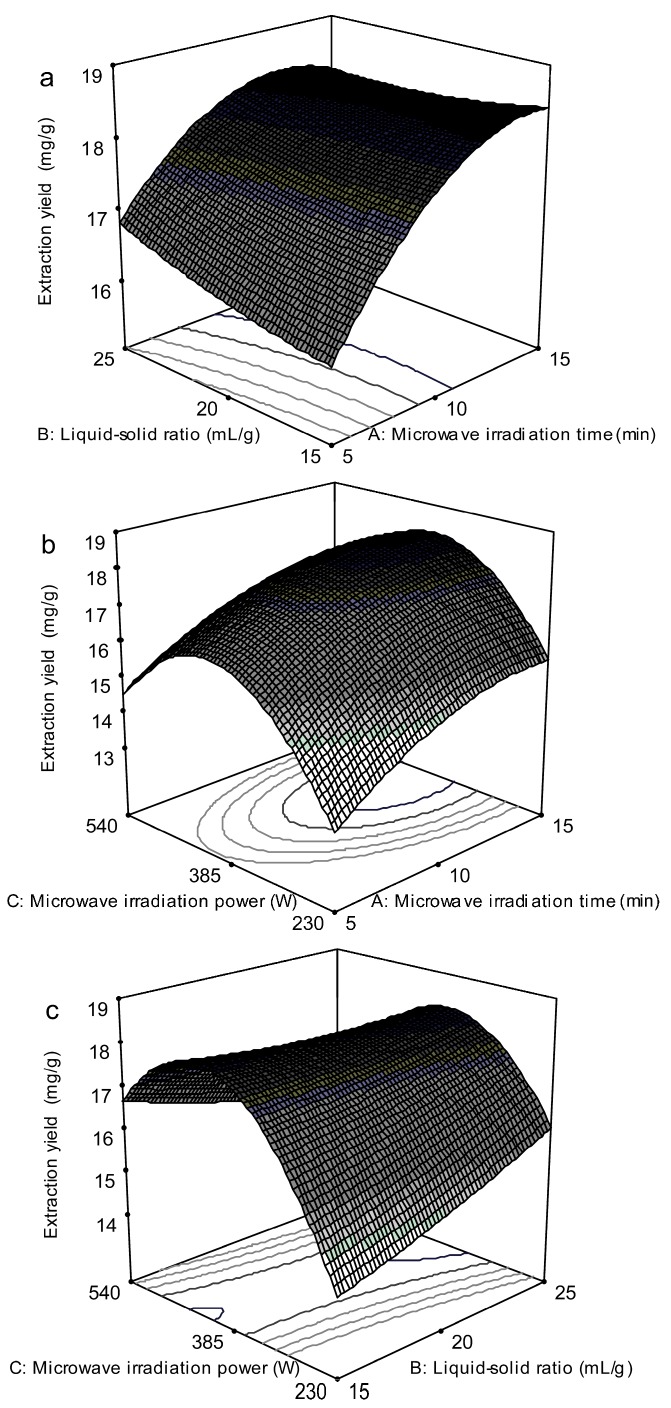
Response surface plots showing the effects of variables on extraction yield of taxifolin. (**a**)Interaction of microwave irradiation time and liquid-solid ratio; (**b**) Interaction of microwave irradiation time and microwave irradiation power; (**c**) Interaction of liquid-solid ratio and microwave irradiation power.

To obtain the optimal levels of the variables for the extraction yield of taxifolin, the 3D surface plots were constructed according to Equation (1). Figure 4a was obtained at the fixed microwave irradiation power of 385 W. The extraction yield increased with the liquid-solid ratio and increased microwave irradiation time, and the maximum extraction yield was obtained at a microwave irradiation time of 14.36 min and liquid-solid ratio of 15:1 mL/g. Figure 4b shows the effect of the microwave irradiation power and time on the taxifolin yield at a fixed liquid-solid ratio of 20:1 mL/g. At a definite microwave irradiation power, the extraction yield increased obviously with the increase of the microwave irradiation time, and nearly reached a peak at the highest microwave irradiation time tested. However, the microwave irradiation power showed a quadratic effect on the response (extraction yield), and the maximum extraction yield was obtained at 393.63 W, followed by a decline with the further increase of the microwave irradiation power. Figure 4c shows the interaction between the microwave irradiation power and liquid-solid ratio at a fixed microwave irradiation time of 10 min. The results indicated that liquid-solid ratio displayed a linear effect on the extraction yield. The quadratic effect of the microwave irradiation power was striking, and the extraction yield reached the highest value at 373.52 W.

**Table 2:** Test of significance for the regression coefficient^a^.

Source	Sum of Squares	Degree of Freedom	Mean Square	*F*-Value	*p*-Value
Model ^b^	41.39	9	4.60	29.21	<0.0001
*X_1_*	7.08	1	7.08	44.97	0.0003
*X_2_*	0.15	1	0.15	0.94	0.3637
*X_3_*	1.58	1	1.58	10.02	0.0158
*X_1_ X_2_*	0.25	1	0.25	1.58	0.2494
*X_1_ X_3_*	0.24	1	0.24	1.53	0.2561
*X_2_ X_3_*	2.67	1	2.67	16.93	0.0045
*X_1_* ^2^	1.97	1	1.97	12.50	0.0095
*X_2_* ^2^	0.051	1	0.051	0.33	0.5860
*X_3_* ^2^	26.60	1	26.60	168.95	<0.0001
Residual	1.10	7	0.16		
Lack of fit	0.45	3	0.15	0.93	0.5024
Pure error	0.65	4	0.16		
Cor total	42.49	16			

^a^: The results were obtained with Design Expert 8.0 software; ^b^: *X_1_* is the microwave irradiation time (min), *X_2 _* is the liquid–solid ratio (mL/g), and *X_3_* is the microwave irradiation power (W).

**Table 3:** Credibility analysis of the regression equations.

Index Mark ^a^	Taxifolin
Standard deviation	0.40
Mean	16.51
Coefficient of variation %	2.40
Press	8.28
*R* ^2^	0.9741
Adjust *R*^2^	0.9407
Predicted *R*^2^	0.8052
Adequacy precision	17.440

^a^: The results were obtained with the Design Expert 8.0 software (Stat-Ease Inc., Minneapolis, MN, USA).

The optimum conditions predicted by the RSM software were: microwave irradiation time 14.09 min, liquid-solid ratio 15.08:1 mL/g, and microwave irradiation power 406.00 W. Under the predicted conditions, the extraction yield of taxifolin can reach 18.54 mg/g. Verification tests were carried out three times under the point prediction conditions (microwave irradiation extraction 14 min, liquid-solid ratio 15:1 mL/g, and microwave irradiation power 406 W) by RSM. The final extraction yield of taxifolin was 18.63 mg/g with a relative error about 1.4%.

### 2.4. Method Validation

#### 2.4.1. Stability

Stability at the optimum conditions obtained was tested by subjecting standard taxifolin (at three concentration levels, 4.13, 8.27, 16.66 g/mL, dissolved in 1.00 M [C_4_mim]Br) to microwave irradiation for 14 min at 406 W microwave power. The recovery of taxifolin was taken as an indicative marker for of the stability of taxifolin at the obtained operating extraction conditions. Data are shown in Table 4. The results indicated that the average recoveries under the operating extraction conditions were 98.79%, 99.03%, and 99.28%, with no change in the retention time of taxifolin. Therefore degradation is insignificant under the selected optimum conditions. The standards in 1.00 M [C_4_mim]Br solution were stored for 7 days. After 7 days, the average recoveries of taxifolin were 96.13%, 97.10%, and 98.80%, respectively.

**Table 4:** Stability studies of taxifolin under the following microwave-assisted extraction conditions.

Initial Concentration (mg/mL)	Recovered Concentration after MAE (mg/mL)	RSD% ( *n* = 3)	Recovery (%)	Recovered Concentration after 7 Days (mg/mL)	RSD% ( *n* = 3)	Recovery (%)
4.13	4.08	1.06	98.79	3.97	1.03	96.13
8.27	8.19	1.00	99.03	8.03	1.07	97.10
16.66	16.54	1.02	99.28	16.46	1.05	98.80

#### 2.4.2. Recovery

Under the above optimized conditions, three samples of larch wood powder with added taxifolin as shown in Table 5 were analyzed. The average recovery of taxifolin was 99.22%.

**Table 5:** Recovery of taxifolin from dried xylem samples of larch (*n* = 3).

Sample	Taxifoin Content of the Sample (mg)	Mass of Added Taxifolin Standard (mg)	Mass of the Sample Analyzed with Added Taxifolin Standard (mg)	Recovery (%)
1	18.67	10	28.61	99.79
2	18.67	20	38.04	98.37
3	18.67	30	48.43	99.51
Average				99.22

#### 2.4.3. Repeatability

Five extraction solutions of the larch wood powder samples were prepared under the optimum conditions of the ILMAE method to assess its repeatability. The average extraction yield of taxifolin showed good repeatability with 0.96% of RSD. The results suggested that taxifolin was stable in the ionic liquid solution and in the extracts. These method validation studies indicated that the proposed method is credible.

### 2.5. Comparison of ILMAE with the Reference Solvent and Traditional Methods

**Figure 5:**
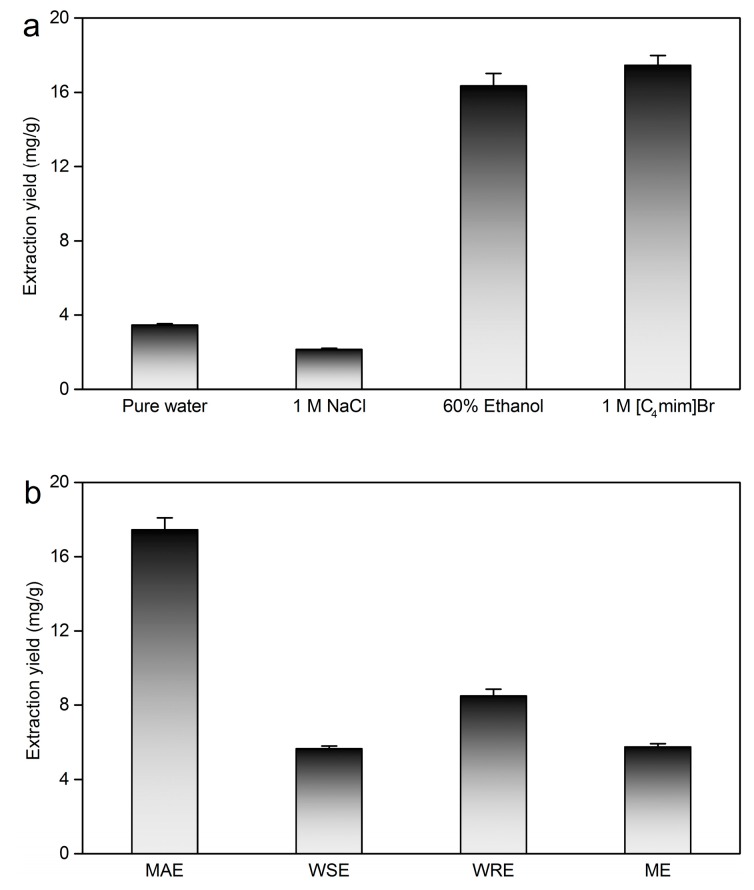
Comparison of ILMAE with the reference solvent and traditional methods. (**a**) Comparison of [C_4_mim]Br with reference solvents for the microwave-assisted extraction of taxifolin; (**b**) Comparison of ILMAE with WSE, WRE, and ME.

In order to further investigate the influence on taxifolin extraction with ionic liquids, traditional solvents (pure water, 1.00 M NaCl and 60% volume fraction ethanol [8,36,37]) were applied to compare with 1.00 M [C_4_mim]Br in microwave-assisted extraction. As shown in Figure 5a, 1.00 M [C_4_mim]Br showed higher efficiency for the extraction of taxifolin than traditional solvents. The main contribution of 1.00 M [C_4_mim]Br to improve taxifolin extraction yield was that ionic liquid possesses unique characteristics compared with water in the whole extraction system. Meanwhile, it can be seen that ionic liquid demonstrated a positive influence on taxifolin extraction. Hence, using ionic liquids to extract taxifolin was superior to traditional solvents.

As shown in Figure 5b, ILMAE was more efficient than traditional taxifolin extraction methods. Pure water is the most common and inexpensive solvent and it is often chosen as the co-solvent in various extraction procedures. WRE is a time-saving but high energy-consumption extraction process. The extraction yield of taxifolin with WRE 4 h was 8.50 mg/g. However, the extraction yield of taxifolin with WSE for 8 h at 50 °C was only 5.64 mg/g, which was similar to ME under the condition of 60% volume fraction ethanol at room temperature for 24 h (5.74 mg/g). Compared with traditional methods, ILMAE exhibited remarkable advantages for taxifolin extraction.

### 2.6. Application and Benefits of the Proposal Approach for Extraction of Taxifolin

The combination of the advantages of microwave irradiation technique with the unique properties of ionic liquids for the extraction of bioactive compounds from plant materials has developed very rapidly in recent years, and has been successfully used in separation of polyphenols [1,19,31,38], aromatic polymers [17,22], flavonoids [18,39], and alkaloids [27]. In this study, good stability, high recovery, and good repeatability of taxifolin yields were observed during the ILMAE extraction procedure which indicated that the proposed method is very credible. This shows the great promise of the proposed ILMAE approach, which could be employed in the field of separation science and analytical science.

### 2.7. Actual Sample Determination

**Figure 6:**
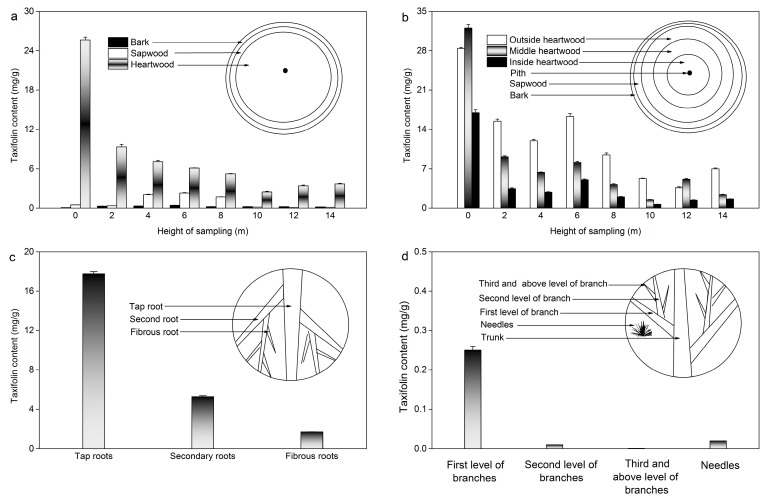
Taxifolin content in different parts of larch*.* (**a**) Taxifolin content in the bark, sapwood, and heartwood; (**b**) Taxifolin content in outside heartwood, middle heartwood and inside heartwood; (**c**) Taxifolin content in roots; (**d**) Taxifolin content in larch branches and needles.

**Figure 7:**
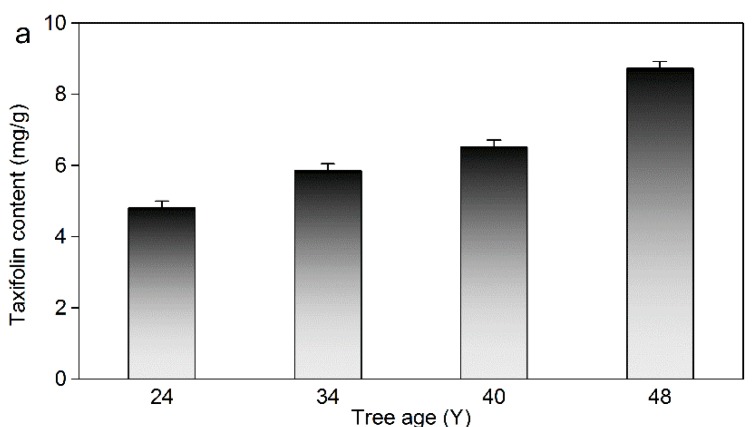

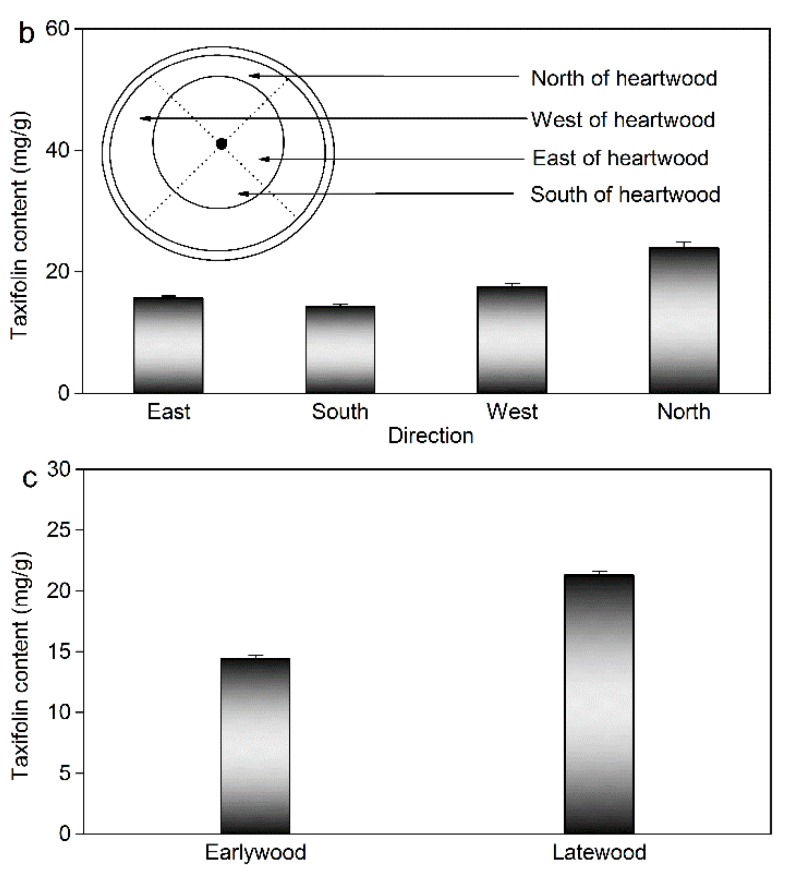
(**a**) The influence of age on taxifolin content; (**b**) The influence of direction on taxifolin content; (**c**) The influence of earlywood and latewood on taxifolin content.

**Figure 8:**
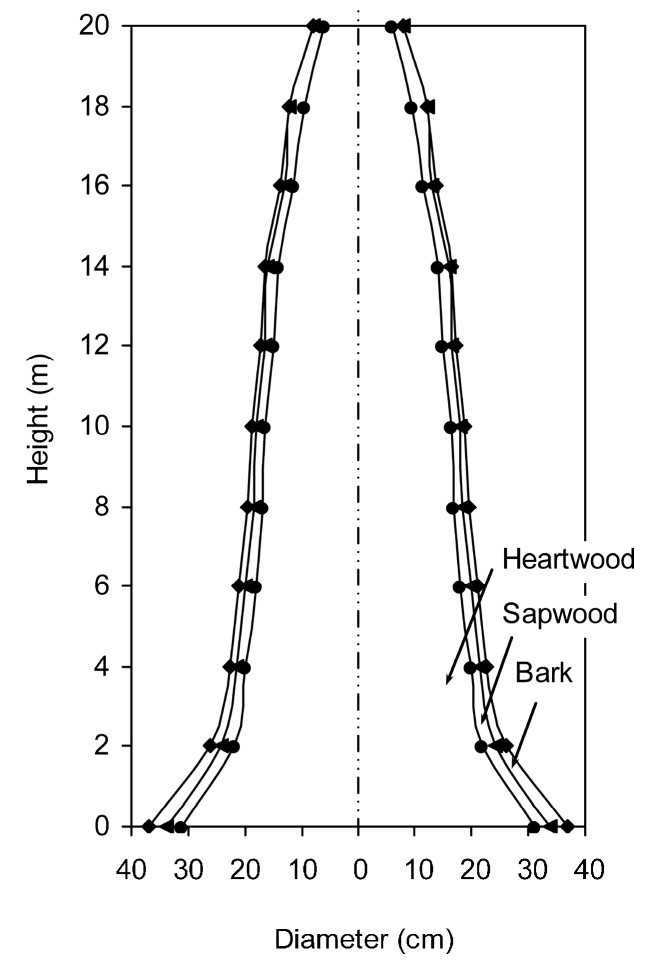
Biomass-related information of larch trunk.

In order to utilize larch resources sufficiently, the taxifolin contents in different parts of larch and the influence of age, orientation, and season were investigated under the optimum ILMAE conditions. The results are summarized in Figure 6 and Figure 7. In addition, biomass-related information is shown in Figure 8.

#### 2.7.1. Taxifolin Content in Bark, Sapwood and Heartwood

As shown in Figure 6a, taxifolin contents in bark, sapwood and heartwood at different heights (0, 2, 4, 6, 8, 12, 14 m) were determined. Compared with larch bark and sapwood at the same height, the content of taxifolin in the heartwood was highest, especially at the height of 0 m (28.32 mg/g). It also manifested that most of the taxifolin was contained in the larch heartwood. Meanwhile, Figure 8 shows that larch heartwood occupies a larger proportion of the biomass than bark and sapwood. Considering both taxifolin content and biomass, heartwood is the best source of taxifolin in larch wood. Hence, it is of great value to study the distribution of taxifolin in larch heartwood. In order to evaluate the content of taxifolin in the heartwood more comprehensively, the heartwood was divided into three parts (outside heartwood, middle heartwood and inside heartwood). As shown in Figure 6b, the content of taxifolin in the middle heartwood at the height of 0 m was higher than in other parts. Meanwhile, with the increasing height of the heartwood, the taxifolin content changed obviously and all are much lower than that at 0 m height.

#### 2.7.2. Taxifolin Content in Roots, Branches and Needles

As shown in Figure 6c, the order of taxifolin distribution in larch root is as follows: tap root > secondary root > fibrous root, which indicated that tap root accounts for the largest amount of taxifolin in larch root. The content of taxifolin in the branches and needles were determined simultaneously. Figure 6d shows that the first branch level possesses a larger amount of taxifolin than the other branch levels or larch needles.

#### 2.7.3. Influence of Tree Age

The trees which were selected for the experiment represented a wide girth range and the age of each *L. gmelinii* was calculated by counting the annual growth rings [40]. The content of taxifolin at different ages (24, 30, 34 and 48 years old) was analyzed in the experiments. As shown in Figure 7a, the taxifolin content increased dramatically with the increasing tree age. According to this result, it can be supposed that the increasing growth age is beneficial for the accumulation of taxifolin in larch.

#### 2.7.4. Influence of Orientation

The taxifolin contents in larch at different orientations were also measured as shown in Figure 7b. Compared with other orientations, taxifolin content on the north side of larch was higher than at other orientations. This indicated that a north orientation is beneficial for taxifolin accumulation.

#### 2.7.5. Influence of Seasons

To know the influence of seasons (mainly focused on earlywood and latewood) on the content of taxifolin, earlywood and latewood were assayed and the results are shown in Figure 7c. The content of taxifolin in earlywood and latewood were 14.42 mg/g and 21.49 mg/g, respectively. This indicated that latewood possesses a higher amount of taxifolin than earlywood. Hence the slow growth rate of larch is conducive to taxifolin accumulation in larch.

## 3. Experimental Section

### 3.1. Chemicals and Reagents

Reference taxifolin (purity ≥ 95%) was obtained from Ametis Company of Blagoveschensk (Amur, Russia). Ionic liquids ([C_2_mim]Br, [C_4_mim]Br, [C_6_mim]Br, [C_8_mim]Br, [C_2_mim]BF_4_, [C_4_mim]BF_4_, [C_6_mim]BF_4_, [C_8_mim]BF_4_, [C_4_mim]Cl, [C_4_mim]TSO, [C_4_mim]NO_3_, [C_4_mim]CIO_4_ and [C_4_mim] HSO_4_, where C_2_mim = 1-ethyl-3-methylimidazolium, C_4_mim = 1-butyl-3-methyl-imidazolium, C_6_mim = 1-hexyl-3-methylimidazolium, C_8_mim = 1-octyl-3-methylimidazolium) were purchased from Chengjie Chemical Co. Ltd. (Shanghai, China) and used without further purification. Acetonitrile and acetic acid of HPLC grade were purchased from J&K Chemical Ltd. (Beijing, China). All the other analytical grade solvents and chemicals were bought from Beijing Chemical Reagents Co. (Beijing, China). Deionized water was purified by a Milli-Q Water Purification system (Millipore, Waltham, MA, USA). All solutions and samples prepared for HPLC analysis were filtered through 0.45 μm nylon membranes.

### 3.2. Materials

*L. gemelinii* samples were provided by the Maoershan Experimental Forest Farm of Northeast Forestry University (Heilongjiang, China) and authenticated by Prof. Wenjie Wang from the Key Laboratory of Forest Plant Ecology, Ministry of Education, Northeast Forestry University, Harbin, China. Their average heights and average diameters were 18.3 m and 17.2 cm at the breast height, respectively. On March, 2012, three trees of each age (24, 34, 40, and 48 years old) were sampled as the raw material of the experiments, and then segmented into many disks from bottom to top. Larches (48 years old) were chosen to systemically evaluate the taxifolin content in different parts. A series of 5 cm high disks (plus or minus 2.5 cm of each height) were sampled for the investigation of taxifolin content at different heights (0, 2, 4, 6, 8, 10, 14 m). Meanwhile, 5 cm high disks of the diameter at breast height were averagely transected into two parts, choosing the heartwoods (below 1.4 m) for the determination of taxifolin content in different orientations and the heartwoods (above 1.4 m) for the analysis of taxifolin content in earlywood and latewood. The results of the detailed sampling methods which also include the root, branches, and needles were plotted as shown in Figure 6 and Figure 7. Each part was dried at ambient temperature, powdered to a homogeneous size and then sieved (60–80 mesh). Wood powder was stored in closed desiccators before use and the same batch of samples were used in the present study. Moisture content of powder was determined before use.

### 3.3. Apparatus

The whole system employed for the extraction of taxifolin was placed in a microwave oven (WP700L20, Galanz Company, Foshan, China) operating at 2450 MHz. The range of microwave power can be continuously adjusted with a maximum output power of 700 W. The oven was modified with the addition of a water condenser and the walls were covered with polytetrafluoroethylene to prevent microwave leakage. The device worked at atmospheric pressure and the maximum power cannot exceed 700 W [25].

### 3.4. HPLC Analysis and Quantification

**Figure 9:**
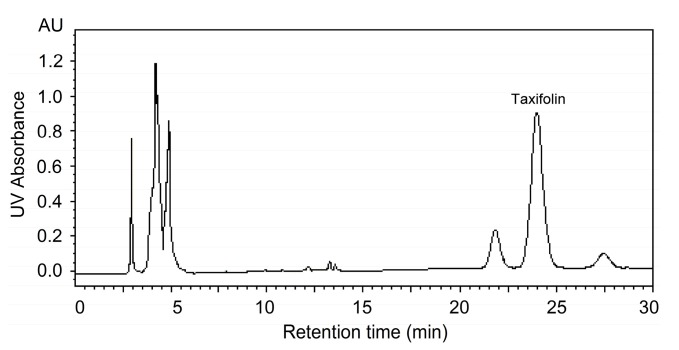
HPLC profile of taxifolin in the [C_4_mim]Br contained extract.

The HPLC system was made up of a Waters 717 automatic sample handling system which consisted of a HPLC system equipped with 1525 Bin pump, 717 automatic column temperature control box and 2487 UV-detector (Waters, Milford, MA, USA). Chromatographic separation was performed on a HiQ sil-C18 reversed-phase column (4.6 mm × 250 mm, 5 μm, Kya Tech, Tokyo, Japan) for the determination of taxifolin. For HPLC analysis, acetonitrile-water-acetic acid (18:81.9:0.1, v/v/v) was selected as the mobile phase with a 1.0 mL/min flow rate, 10 μL injection volume, and 25 °C column temperature. As shown in the Figure 9, taxifolin was determined at a wavelength of 294 nm, 30 min run time and 24 min retention time. It was resolved sufficiently to give baseline separation. Taxifolin content in different parts of larch was also identified by comparing the retention time with that of a standard solution. The corresponding calibration curve equation for taxifolin was *Y* = 2.9480 × 10^7^ *X* + 1.5121 × 10^5^ (r = 0.9999). Good linearity was found for the determination of taxifolin in the range from 0.0625 to 1.0000 mg/mL. The extraction yield or content were expressed as milligrams of taxifolin per gram dry mass of samples.

### 3.5. Ionic Liquids Based Microwave-Assisted Extraction

Dried larch wood powder (0.5 g) was mixed with various ionic liquid aqueous solutions in a 50 mL flask, and then the suspensions were irradiated under microwave heating. The conditions for the extraction of taxifolin, including cations and anions of the ionic liquids, ionic liquid concentration, soaking time, liquid-solid ratio, and microwave irradiation power and time, were systematically optimized to achieve a satisfactory extraction yield. After extraction, the obtained extracts were cooled to ambient temperature and then analyzed by HPLC. The mean mass of the wood powder was the average mass of three samples before extraction.

### 3.6. Optimization ILMAE by RSM

The Design Expert (Version 8.0, Stat-Ease Inc., Minneapolis, MN, USA) software was used for experimental design, data analysis, and model building. A Box-Behnken design (BBD) was employed for optimization with respect to three important variables the microwave irradiation time, liquid-solid ratio, and microwave irradiation power. The factorial design consists of twelve factorial points and five center points. Extraction yield of taxifolin was chosen as the response for the combination of the independent variables given in Table 1. Each condition was carried out three times and the mean values were expressed as determined values. Experiments were carried out randomly to minimize the influences of unexpected variability in the determined values. A quadratic equation was used for this model as follows:

(2) $$Y=\beta_{0}+\sum_{i=1}^{3}\beta_{i}X_{i}+\sum_{i=1}^{3}\beta_{ii}X_{i}^{2}+\sum_{i=1}^{2}\sum_{j=i+1}^{3}\beta_{ij}X_{i}X_{j}$$

where *Y* is the estimated response; β*_0_*, β*_i_*, β*_ii_*, and β*_ij_* are the regression coefficients for intercept, linearity, square, and interaction, respectively; and *X*_1_, *X*_2_, and *X*_3_ are the independent variables.

### 3.7. Method Validation

Stability tests were performed using taxifolin standard dissolved in 1.00 M of [C_4_mim]Br by ILMAE under the optimum conditions. The recovery of taxifolin was taken as indicative of the stability of taxifolin at the derived operating extraction conditions. To determine the repeatability of the novel extraction method, five samples of the same weight (0.5 g) were processed under the same optimum extraction conditions as those obtained from the systematic study of the different extraction parameters.

### 3.8. Reference and Conventional Extraction Methods

Pure water, 1.00 M sodium chloride, and 60% volume fraction of ethanol were chosen as reference solvents for microwave-assisted extraction of taxifolin from larch wood powder*.* Experiments were carried out under the optimized conditions (microwave irradiation time 14 min, liquid-solid ratio 15:1 mL/g and microwave irradiation power 406 W) by the microwave-assisted extraction method. Extracts were cooled to ambient temperature and filtered to be analyzed by HPLC. The conventional extraction methods of taxifolin include WSE, WRE, and ME. WSE conditions involved heated for 8 h at 50 °C with water, WRE conditions is microwave irradiation for 4 h with water, and ME conditions is immersing larch wood powder in 60% volume fraction of ethanol at room temperature for 24 h. The extraction yield of taxifolin with different kinds of extraction methods were compared and analyzed.

### 3.9. Statistical Analysis

The ANOVA test was used to calculate the significance of the differences of taxifolin extraction yield. The results of HPLC analysis were expressed as means of extraction yield ± SD.

## 4. Conclusions

In this study, we have come up with a novel ILMAE for extraction of taxifolin from *L. gmelinii*. In consideration of the influence of both the anion and cation of ionic liquids, [C_4_mim]Br was selected for the subsequent analysis. The microwave-assisted extraction conditions were optimized using single factor experiments and BBD in detail. Under the abovementioned conditions, a satisfactory taxifolin extraction yield was obtained. Compared with traditional solvents and methods, ILMAE provided higher extraction yield, shorter time and energy consumption. Meanwhile, the distribution of taxifolin in different parts of larch and the influences of age, orientation, and season on taxifolin accumulation were also evaluated in this work for the utilization of the larch resources, which not only demonstrated that larch heartwood possesses the largest amount of taxifolin as well as biomass, but also showed that increasing plant age, north orientation and slow growth rate are beneficial for taxifolin accumulation in larch.
